# Niche formation and function in developing tissue: studies from the *Drosophila* ovary

**DOI:** 10.1186/s12964-022-01035-7

**Published:** 2023-01-27

**Authors:** Jian Jin, Ting Zhao

**Affiliations:** 1 School of Educational Science, Anhui Normal University, Wuhu, 241000 People’s Republic of China; 2 School of Life Science, Hubei Key Laboratory of Genetic Regulation and Integrative Biology, Central China Normal University, Wuhan, 430079 People’s Republic of China

**Keywords:** Niche, *Drosophila*, Hippo, Insulin, Ovary

## Abstract

Adult stem cells have a unique ability to self-renew and to generate differentiated daughter cells that are required in the body tissues. The identity of adult stem cells is maintained by extrinsic signals from other cell types, known as niche cells. Thus, the niche is required for appropriate tissue homeostasis. Niche is formed and recruits stem cells during tissue development; therefore, it is essential to establish niche cells and stem cells in proper numbers during development. A small niche may recruit too few stem cells and cause tissue degeneration, while a large niche may maintain too many stem cells and lead to tumorigenesis. Given that vertebrate tissues are not suitable for large-scale forward genetics studies, the *Drosophila* ovary stands out as an excellent model for studying how multiple niche cell types and germ cells (GCs) are coordinately regulated in vivo. Recent studies are beginning to reveal how various signaling molecules regulate niche formation and how niche cells non-autonomously influence GC number. In this review, we summarize the ovarian niche structure, the key signaling pathways for niche formation, and how niche cells generate extrinsic factors to control GC proliferation during ovarian development.

**Video abstract**

**Supplementary Information:**

The online version contains supplementary material available at 10.1186/s12964-022-01035-7.

## Introduction

Stem cells and their niches constitute functional units that maintain body tissue homeostasis [1–4]. In 1978, the concept of the niche was first proposed by Schofield [5] to describe the physiological microenvironment that supports stem cells; since then, niche structures have been identified in a host of groups, from invertebrates to mammals [6–10]. A stem cell niche allows the establishment of stem cells and the maintenance of a balance between stem cell self-renewal and differentiation [11–14]. Many studies have focused on stem cell maintenance in the adult stage [15–18]. The niche is established and anchors stem cells during tissue development [19]; therefore, it is essential to establish niche and stem cells in suitable numbers during development. However, very little is understood about how to form fixed numbers of both niche cells and stem cells during development. Understanding the underlying regulatory mechanisms behind this process will help to identify genetic developmental defects that disrupt adult tissue formation.

The mammalian ovary is not appropriate for large-scale systematic screening of genes involved in niche formation and function; however, *Drosophila melanogaster* overcomes this limitation. In this context, the *Drosophila* ovary provides an effective model for studying the genetic and molecular mechanisms behind niche formation and function during ovary development. Herein, we discuss recent knowledge on how niche formation is regulated by signaling molecules and how stem cell number is controlled by extrinsic factors coming from the niche.

## Formation of the *Drosophila* ovarian niche

**Fig. 1:**
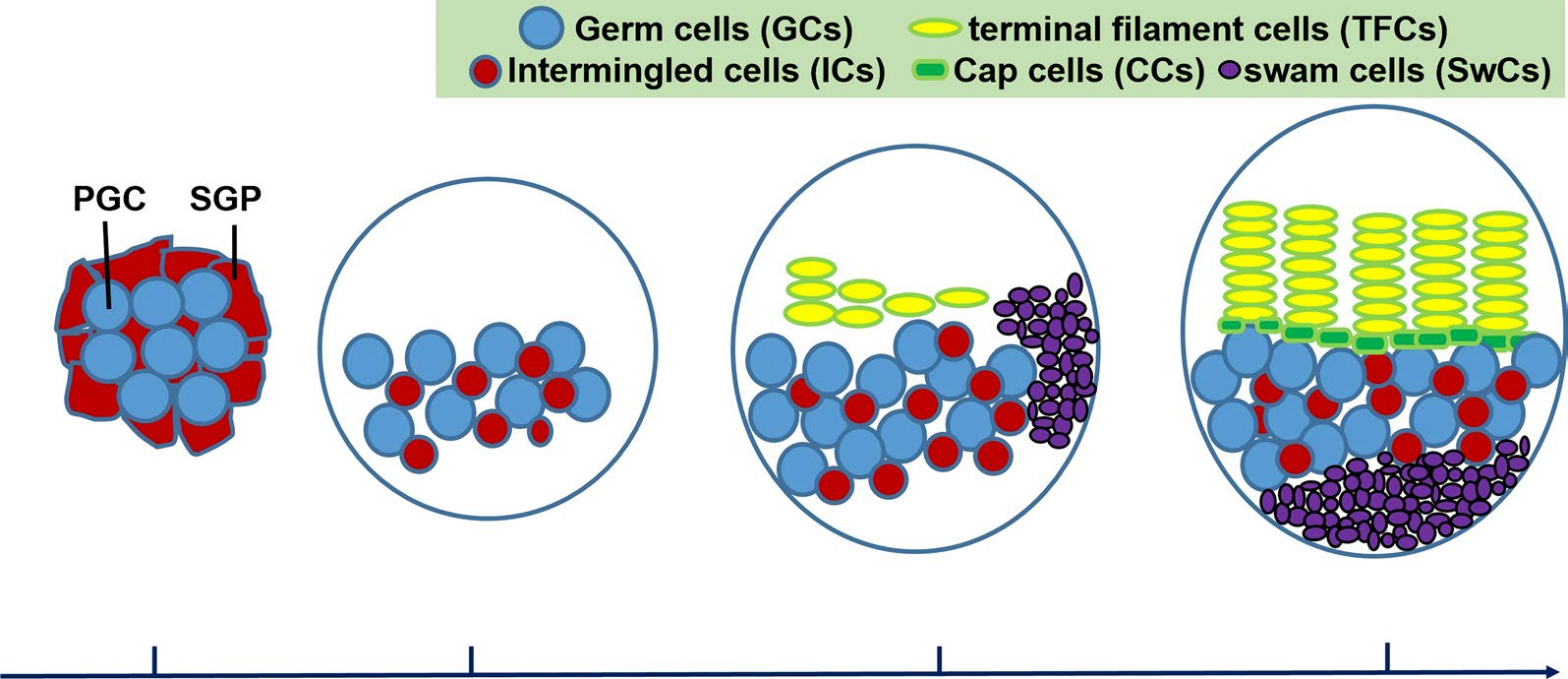
Schematic of ovarian development in *Drosophila* from the embryo to the larval–pupal (LP) transition stage. The embryonic gonad consists of primordial germ cells (PGCs: blue) and somatic gonadal precursors (SGPs: red). The L2 larval ovary is composed of germ cells (GCs: blue) surrounded by intermingled cells (ICs: red). By the mid-third instar larval (ML3), terminal filament cells (TFCs: yellow) first appear and somatic swarm cells (SwCs: purple) are located dorsolaterally. During the LP transition, SwCs form a new posterior domain and the ICs adjacent to basal TFCs differentiate into cap cells (CCs: green); the TFCs are organized into 16–20 TF stacks

The embryonic ovary in *Drosophila* consists of two primary cell types: the primordial germ cells (PGCs) and the somatic gonadal precursors (SGPs) (Fig. 1) [20, 21]. During early larval stages, PGCs, which are the precursors of GCs, and somatic precursors proliferate [22]. In the second instar larval (L2) stage, somatic cell-derived intermingled cells (ICs) occupy the central region of the larval gonad and closely interact with GCs (Fig. 1) [23]. By the mid-third instar larval (ML3), somatic swarm cells (SwCs) are located dorsolaterally [24]. Also, terminal filament cells (TFCs) first appear and start to proliferate (Fig. 1) [25, 26]. After 24 h, these TFCs finish flattening, sorting, intercalation and stacking, and finally form 16–20 regularly arranged terminal filaments (TFs) [26]. By that time, most SwCs have also completed their movements and have formed a new posterior domain (Fig. 1) [24]. During the larval–pupal transition (LP transition), ICs adjacent to the basal TFCs differentiate into cap cells (CCs) [27, 28]. Although TFCs and CCs are adjacent to each other, they can be distinguished by morphologies: TFCs are cuboid-shaped whereas CCs are disc-shaped [26, 29]. Once TFs and CCs are formed, GCs can attach to them to become adult GSCs. At the prepupal stage, the newly formed GSC niche, composed of somatic cell TFCs, CCs, and ICs, becomes functional [28].

The availability of enriched genetic tools, including many cell type-specific Gal4 drivers (Table 1) for performing targeted gene knockdown, rescue, or overexpression manipulation in niche cells makes *Drosophila* ovarian GSC niche an attractive system for studying niche formation and function.

**Table 1:** Cell type-specific Gal4 drivers in the ovarian niche of *Drosophila*

Gal4 name	Cell-type expression	References
Tj-Gal4	Intermingled cells and cap cells	[23]
hh-Gal4	Terminal filament and cap cells	[30]
bab1-Gal4	Terminal filament and cap cells	[31]
c587-Gal4	Intermingled cells	[15]
en-Gal4	Terminal filament and cap cells	[32]

## Signal regulation during the formation of the ovarian niche

The ovarian niche consists of three different types of somatic cells. These cells start to proliferate at different times and several different signaling pathways are involved in this process. Below, we summarize the signaling pathways that regulate the formation of CCs, TFCs, and ICs.

## Pathways that regulate CC formation

Activation of Notch signaling helps to promote CC formation [29, 33]. Xie and colleagues showed that newly formed TFCs express the Notch ligand Delta, which activates Notch signaling in adjacent somatic cells and induces them to become CCs [29]. Overexpression of Delta or forced expression of activated Notch results in an increase in CC number [29, 33]. Another key signaling pathway that plays a role in niche formation is related to Ecdysone signaling [22, 34]. Reduction of Ecdysone signaling activity in somatic cells results in expansion of CCs [34]. Moreover, Ecdysone prevents precocious CC differentiation. In *Ecdysone Receptor* (*EcR*) RNAi ovaries, CCs appear at ML3 instead of the prepupal stage [22]. Given that both the Notch and Ecdysone signaling pathways are involved in CC formation, it is important to know whether and how these two pathways interact to regulate the CC formation process. Yatsenko and Shcherbata explored this question and found that *miR-125* acts as an intermediary between Notch and Ecdysone signaling in the process of CC formation [35]. Specifically, Ecdysone signaling induces expression of *miR-125*, which targets a Notch signaling antagonist called Tom. Downregulation of Tom activates Delta, which activates Notch signaling in adjacent somatic cells and converts them into CCs [35].

In addition to the Notch and Ecdysone signaling pathways, protein Traffic jam (Tj) affects the specification of CC [27], but whether it affects CC number is not yet clear.

## Pathways that regulate TFC formation

TFC proliferation is controlled by several signaling pathways, including the Notch, Hippo, Janus kinase/signal transduction and activator of transcription (JAK/STAT), Insulin and Target of rapamycin (Tor) pathways (Table 2) [36–40]. Yatsenko and Shcherbata showed that Notch signaling is required for TFC formation. In *Notch* mutant ovaries, TFs are abnormally shaped and TFC number is reduced, indicating that Notch signaling is essential for TF morphogenesis and TFC formation [36]. Hippo signaling pathway components Hippo (Hpo) and Yorkie (Yki) are both expressed in TFCs [37]. Altering Hippo pathway activity in soma notably affects TFC proliferation and TF stack formation: RNAi of *hpo* or *warts* (*wts*) or overexpressing *yki* significantly increases TFC and TF stack number. Conversely, knocking down *yki* or overexpressing *hpo* in the soma results in a remarkable decrease in TFC and TF stack number [37]. In addition, knocking down *hpo* in somatic cells leads to notably increased Stat92E expression in TFCs and ICs, suggesting that Hippo pathway activity limits JAK/STAT pathway activity in the soma, as Stat92E is a readout of JAK/STAT activity [37]. In addition, decreasing the activity of Dome, a JAK/STAT receptor, in the soma significantly decreases TFC number while double knockdown of *dome* and *hpo* completely suppresses the phenotype of *hpo* knockdown alone [37]. This genetic evidence suggests that Hippo signaling regulates TFC proliferation through interactions with the JAK/STAT pathway.

**Table 2:** Summary of changes in TFC, IC, and GC numbers when the expression levels of genes from different signaling pathways are altered in somatic cells

Genotype	Average number of TFs	Average number of ICs	Average number of GCs	Refs
tj-Gal4	19.5 ± 2.6	466.7 ± 124.9	140.7 ± 23.7	[37]
tj > hpo RNAi	25.5 ± 2	818.2 ± 124.5	267.2 ± 24.3	[37]
tj > wts RNAi	23.6 ± 3.7	796.1 ± 168	271.5 ± 57.9	[37]
tj > yki RNAi	17.1 ± 3	322.5 ± 83.6	157.7 ± 43.2	[37]
tj > UAS-yki	26 ± 4.4	1186.9 ± 382.7	329.5 ± 23.7	[37]
tj > UAS-hpo	15.3 ± 2.1	301.6 ± 40.8	166.6 ± 35.3	[37]
tj > LacZ	18.34 ± 2.3	341.08 ± 50.5	132.98 ± 19.12	[38]
tj > InR	49.86 ± 10.22	1432.54 ± 285.66		[38]
tj > Akt RNAi	1.03 ± 1.32	118.77 ± 68.36	40.94 ± 9.89	[38]
tj > Tor RNAi	5.38 ± 1.19	149.82 ± 21.41	61.92 ± 15.83	[38]
tj > chico RNAi	5.36 ± 2.57	213.5 ± 21.44	85.17 ± 15.83	[38]
c587-Gal4		344 ± 35	97 ± 13	[52]
c587 > EgfrDN		89 ± 17	204 ± 8	[52]
c587 > EgfrCA		352 ± 30	57 ± 12	[52]
nos > spi RNAi		299 ± 27	122 ± 25	[52]

Nutritional cues provided by the Insulin and Tor pathways also affect TF number (Table 2) [38–41]. In *Drosophila*, Insulin binding to the Insulin-like receptor (InR) induces recruitment and phosphorylation of the Insulin receptor substrate (encoded by the *chico* gene) and subsequent activation of Phosphoinositide 3-kinase (PI3K) and Akt [42, 43]. Gancz and Gilboa reported that overexpression of *InR* in somatic cells results in a significant increase in TF number. Conversely, knocking down *chico*, *Akt*, or *Tor* by RNAi leads to a remarkable reduction in the number of TFs [38].

Activin signaling is transmitted by Baboon (Babo), a type I receptor of the TGF-β (transforming growth factor-β) superfamily in *Drosophila* [44]. Lengil et al. found that Activin signaling promotes TF formation in developing ovaries [45]. Gancz et al. showed that Ecdysone can also accelerate TF formation [22]. In wild-type ML3 ovaries, only a few short TFs can be detected. In contrast, a significant increase in TF stack number is observed in ovaries of *Babo* mutant and *EcR* knockdown line. In addition, in the wild-type, TFs are well organized, whereas in the ovaries with low activity of Ecdysone or Activin receptor, they are unevenly spaced [22, 45], suggesting that precocious TF formation results in morphogenesis defects.

In addition to these signaling pathways, a few genes, including *twinstar* (*tsr*), *bric-à-brac* (*bab1/bab2*), *LIM homeobox transcription factor 1 alpha* (*Lmx1a*) and *longitudinals lacking* (*lola*), have been reported to be involved in TFC formation [46–50]. Chen et al. found that in *tsr* mutant ovaries, the TFC number decreased and all TFCs have a rounded appearance. Importantly, the TF structure is not present, suggesting that *tsr* is essential for the formation of TFC and TF [46]. In 1995, Godt and Laski discovered that *bab* affects TF formation [25]. The *Bab* locus encodes Bab1 and Bab2. In 2020, a study found that a reduction of both Bab1 and Bab2, but not each separately, impedes TF formation [48]. Another study found that the transcription factor Lmx1a is required for the formation of TF. In *Lmx1a* mutant ovaries, TFCs fail to organize in individualized stacks, leading to aberrant TF structures [49]. That previous study also showed that *bab1/bab2* is essential for *Lmx1a* expression and function, which raised the possibility that *bab1/bab2* affects TF formation by regulating *Lmx1a* expression. However, this requires further confirmation, to answer questions such as whether overexpression of *Lmx1a* can rescue *bab1/bab2* mutant phenotype in vivo. Zhao et al. reported that *lola* is also essential for TF formation. In *lola* RNAi ovaries, either no TF stacks are formed or they are disordered [50]. Coincidentally, both *lola* and *bab/bab2* have a BTB domain, suggesting that this domain may be necessary for TF formation.

## Pathways that regulate IC formation

ICs are in direct contact with GCs and are thought to give rise to escort cells in adult ovaries [22, 29, 51]. The Hippo signaling pathway is involved in IC proliferation [37]. Lowering Hippo pathway activity by knocking down *hpo* or *wts* or overexpressing *yki* results in a remarkable increase in IC number. On the contrary, overexpression of *hpo* or depletion of *yki* by RNAi significantly reduces IC number (Table 2) [37]. Sarikaya and Extavour found that knockdown of *hpo* in the soma significantly increased pMAPK (a readout of epidermal growth factor receptor [EGFR] activity) expression in ICs, suggesting that Hippo activity limits EGFR activity in ICs. Furthermore, knockdown of *hpo* and *egfr* partially rescues *hpo* RNAi-induced overgrowth of ICs [37]. These results suggest that the Hippo pathway interacts with the EGFR pathway to regulate IC number. That study also found that the JAK/STAT signaling activity is very high in ICs, and that RNAi against JAK/STAT receptor *dome* or the ligand *unpaired* (*upd1*) can rescue the increased IC number resulting from *hpo* RNAi. These results suggest that the Hippo pathway interacts with the JAK-STAT and EGFR signals in regulating IC proliferation.

Insulin and Tor signaling play a role in the proliferation of ICs [38]. Somatic overexpression of InR significantly increases IC number. By contrast, RNAi against *Tor*, *chico*, or *Akt* leads to a decrease in IC number (Table 2) [38]. Whether the EGFR pathway regulates IC number is controversial. One study found that blocking Egfr signaling in ICs results in fewer ICs [52]. However, another reported that IC number was unaltered by knockdown of *egfr* or *spi* alone [37].

According to the signaling pathways that regulate the formation of TFs and ICs, it can be noted that the Hippo signaling and Insulin/Tor signaling have the same effect (Table 2). In addition, simultaneous reduction of the Hippo and JAK/STAT pathway activity has the same effect on IC and TFC numbers. Moreover, these studies used Tj-Gal4, a driver that is only expressed in ICs, not in TFCs, to drive gene knockdown or overexpression in soma. Thus, these data indicate that signals in ICs non-autonomously regulate TFC proliferation.

## Niche function: extrinsic cues for GC proliferation

In the *Drosophila* embryonic gonad, there are initially ~ 12 PGCs [53]. During the larval stage, the PGCs remain undifferentiated and proliferate to more than 100 [54, 55]. This proliferation process is regulated by extrinsic signaling molecules, which are released from niche cells.

Signaling molecules in the niche cells regulate GC proliferation non-autonomously (Table 2) [22, 37, 38, 56]. The Hippo pathway regulates the proliferation of GCs: *hpo* or *wts* RNAi or overexpression of *yki* in the soma leads to a significant increase in GC number. On the contrary, overexpression of *hpo* in the soma decreases GC number [37]. Lowering EGFR activity in the soma also increases GC number [52, 57]. Moreover, double knockdown of *hpo*/*egfr* results in fewer GCs, which completely rescues the *hpo* RNAi-induced increase in GC number [37]. In addition, knockdown of JAK/STAT receptor *dome* or the ligand *upd1* completely rescues *hpo* RNAi-induced GC overproliferation [37]. These results indicate that Hippo signaling regulates GC proliferation non-autonomously via interactions with the EGFR and JAK/STAT pathways.

The Insulin and Tor signaling also play a role in GC proliferation [38]. Somatic expression of *Tor*, *chico*, or *Akt* RNAi non-autonomously reduces GC number, and somatic overexpression of *InR* results in precocious GC differentiation [38]. RNAi constructs against *EcR* lead to precocious GC differentiation as well [22]. Recently, Lehmann lab utilized a single-cell RNA atlas of LL3 ovaries and identified the SwCs as mediators of the Ecdysone signal for GC differentiation: once SwCs reach the posterior of the ovary, the Ecdysone induces expression of Torso-like (Tsl) in SwCs, which acts as a soma-to-germline signal to stimulate PGC differentiation [24, 58]. Of note, Gancz and Gilboa found that the Insulin and Ecdysone pathways act in parallel to regulate GC differentiation [38].

It can be noted that Insulin and Ecdysone signaling pathways are required in parallel for GC differentiation and niche formation, which raises an interesting question of how these two different processes are connected.

## Signaling crosstalk in ovarian niche and germ cells

**Fig. 2:**
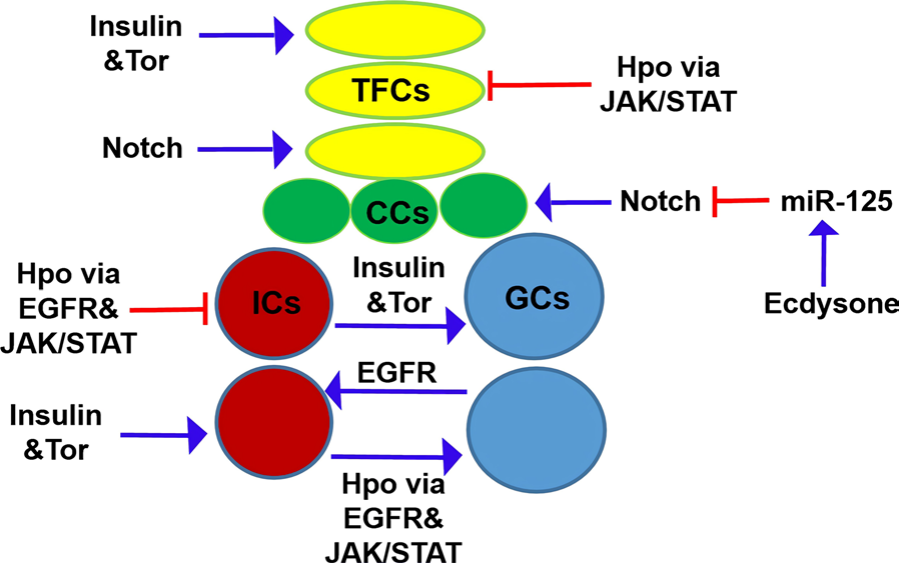
Schematic diagram of signaling crosstalk that regulates the formation of ovarian niche cells and germ cells. The Hippo, Insulin and Tor signaling pathways function in the soma to regulate proliferation both autonomously in TFCs and ICs and non-autonomously in GCs. The Notch signaling is required for TFC and CC formation. The EGFR pathway regulates homeostatic growth of both IC and GC numbers

When organized niches that contain a fixed number of stem cells are established during organ development, the proliferation rate of the niche cells and stem cells should be coordinated. How such coordination is achieved and how systemic factors might affect these processes remain largely unknown. The developing *Drosophila* ovary, which contains proper number of niche cells and germ cells, is an excellent model system with which to investigate this problem. Table 2 and Fig. 2 summarize the available data on the signaling pathways that regulate the formation of niche cells and GCs. The Ecdysone-miR-125-Notch signaling is required for CC formation. The Hippo signaling regulates TFC and GC proliferation through interactions with the JAK/STAT pathway. In addition, the Hippo pathway interacts with the JAK-STAT and EGFR signals to regulate IC proliferation. The Insulin and Tor signaling pathways function in the soma and regulate proliferation, each autonomously in ICs and TFCs and non-autonomously in GCs.

It is noteworthy that GCs can influence the survival of IC conversely [52]. Gilboa and Lehmann found that EGFR functions in ICs to inhibit GC proliferation, and in turn, GCs express Spitz (an EGFR ligand), which is required for IC survival [52]. Thus, coordination of growth between the soma and germ line in the developing ovary is achieved.

## Conclusions and future perspectives

It has been more than 40 years since the concept of the niche was proposed. During this time, we have learned that the function of the niche is to provide extrinsic signals for stem cells to maintain their identity in adult tissue. Stem cells self-renewal is crucial for development and tissue homeostasis in various organisms. For example, in the testes of *Drosophila*, hub cells (stem cell niche) are implicated as a source of JAK-STAT signaling that promotes self-renewal of both GSCs and CySCs (somatic cyst stem cells) [59–61]. Brawley and Matunis showed that loss of Stat92E leads to a loss of all GSCs from the niche [59]. In CySCs, *zinc finger homeodomain 1* (*zfh1*) and *chronologically inappropriate morphogenesis* (*chinmo*) are direct target genes of Stat92E [60, 61]. Flaherty et al. reported that CySCs lacking either *zfh1* or *chinmo* rapidly differentiate [60, 61]. In fact, during tissue development, the niche has already begun to provide growth factors for germ cells. Dennd1a, which is mainly expressed in somatic cells in the ovaries of mouse fetuses, is essential for oogenesis. Its disruption results in a significant reduction in germ cell number [62]. Thus, although the structure of the mammalian ovary is different from that of *Drosophila*, they share a common phenomenon wherein the niche forms and controls GC proliferation during ovary development.

Some studies have reported that some genes (e.g., *tsr*, *bab1/bab2*, *lola*, and *Lmx1a*) affect niche formation, but it is unclear whether these genes function through the known signaling pathways to control niche formation. At present, only one study has demonstrated that GCs can control niche cell survival; whether there are more mechanisms through which GCs regulate proliferation of niche cell remains unknown. Most importantly, how niche cells are specified and how their fates are stabilized remain unclear.

The answers to the above questions will certainly provide a better understanding of how niche and germ cells develop coordinately at the molecular and cellular levels. As stem cells and their niches have many similarities across species, the knowledge gained from the *Drosophila* system will provide insight into niche and stem cell regulation in mammalian systems.

## Data Availability

Not applicable.

## Abbreviations

GCs: Germ cells
PGCs: Primordial germ cells
SGPs: Somatic gonadal precursors
L2: Second instar larval
ML3: Mid-third instar larval
LP transition: Larval–pupal transition
ICs: Intermingled cells
TFCs: Terminal filament cells
TFs: Terminal filaments
CCs: Cap cells
SwCs: Somatic swarm cells
EcR: Ecdysone Receptor
InR: Insulin-like receptor
Tj: Traffic jam
JAK/STAT: Janus kinase/signal transduction and activator of transcription
Tor: Target of rapamycin
EGFR: Epidermal growth factor receptor
Lmx1a: LIM homeobox transcription factor 1 alpha
